# JAK Inhibitors for the Treatment of Axial Spondyloarthritis

**DOI:** 10.31138/mjr.34.2.129

**Published:** 2023-06-30

**Authors:** Kalliopi Klavdianou, Charalampos Papagoras, Xenofon Baraliakos

**Affiliations:** 1 Rheumazentrum Ruhrgebiet Herne, Ruhr-University Bochum, Germany,; 2 Department of Rheumatology, ‘Asklepieion’ General Hospital, Athens, Greece,; 3 First Department of Internal Medicine, University Hospital of Alexandroupolis, Democritus University of Thrace, Alexandroupolis, Greece

**Keywords:** JAK inhibitors, axial spondyloarthritis, ankylosing spondylitis, non-radiographic axial spondyloarthritis

## Abstract

Axial spondyloarthritis (axSpA) is a chronic disease characterized by inflammation and new bone formation that causes pain and results in functional impairment and long-term disability. Biologic agents targeting TNFα or IL-17 have been the mainstay of treatment for patients with axSpA and an inadequate response to nonsteroidal anti-inflammatory drugs. However, a proportion of axSpA patients do not respond adequately to those drugs either, creating the need to target alternative disease pathways. Janus kinase (JAK) inhibitors (JAKis) are a group of targeted synthetic disease-modifying anti-rheumatic drugs that block the intracellular signalling pathway of several proinflammatory cytokines. Given their efficacy in the management of rheumatoid arthritis and that JAKs mediate the signalling of cytokines involved in the pathogenesis of axSpA as well, JAKis have been successfully tested in a number of clinical trials in axSpA, which has led to the approval of two compounds, tofacitinib and upadacitinib for the treatment of the disease. Data from new clinical trials, long-term extensions of completed trials, and real-life observational studies that continuously emerge will shape the efficacy and safety profile and ultimately the place of JAKis in the treatment of AxSpA.

## INTRODUCTION

Janus kinases (JAKs) are a group of intracellular tyrosine kinases with four members in humans [JAK1, JAK2, JAK3, and Tyrosine kinase2 (Tyk2)], involved in the signalling of plenty of cytokines and growth factors.^1^ Upon binding of a cytokine molecule to its cell membrane receptor, a specific combination of JAKs associate with the cytoplasmic domain of the receptor leading both to JAK and to the receptor phosphorylation. This enables the recruitment of transcription factors of the signal transducer and activator of transcription (STAT) family. Each receptor/JAK complex associates with specific STATs which are subsequently activated via phosphorylation by JAKs. Following this step STATs translocate to the nucleus and regulate target gene expression.JAK inhibitors (JAKis) are a relatively new class of drugs that bind and inhibit the enzymatic activity of JAKs. Several JAKis have been developed for a broad array of clinical applications, including immunology and hematology.^2^ Among them, at least 5 compounds have already been licensed for rheumatologic indications across the globe forming the first members of the so-called targeted synthetic disease modifying antirheumatic drugs (tsDMARDs): tofacitinib, baricitinib, upadacitinib, filgotinib, and peficitinib. They are small molecules which can be taken up orally in contrast to the traditional biological DMARDs (bDMARDs). Most literature regarding their mode of action and clinical effects in inflammatory arthritis derive from studies in rheumatoid arthritis (RA), which is the first rheumatic condition those drugs have been approved for. Indeed, in RA JAKis modulate the intracellular signalling of plenty of cytokines within a multi-level and intertwined cytokine network resulting in high levels of disease response, often surpassing TNFα inhibitors.^3,4^ They have also an acceptable safety profile, with some common features across the class, such as an increased risk for herpes zoster.^5^

The pathogenesis of axial spondyloarthritis (axSpA) is multifactorial involving various immune cells and cytokines. Innate immune cells such as dendritic cells, macrophages, mast cells, innate-like lymphocytes (ILCs) and mucosal-associated invariant T cells (MAITs) are activated often at extra-skeletal sites, such as the gut, and release cytokines or migrate themselves to musculoskeletal structures, primarily the entheses.^6,7^ A number of cytokines such as IFNγ, IL-6, IL-12, IL-23, IL-17, and TNFα are involved in this process.^8,9^ Enhanced myelopoiesis and neutrophil priming through GM-CSF also appear to contribute to the SpA-related inflammation.^10,11^ Several of those cytokines including IFNγ, IL-6, IL-12, IL-23 and GM-CSF signal directly through JAKs. On the other hand, although the “signature cytokines” of AxSpA, i.e. IL-17 and TNFα, do not depend on JAK-STAT activation, they are either downstream of JAK-dependent cytokines, such as IL-23, or their effects on target cells are modulated by other cytokines signalling via JAK.^12–15^ Indeed, data from animal models suggest a link connecting JAKis and the IL-23/IL-17 axis and therefore could at least partially explain the efficacy of this drug class in psoriatic arthritis (PsA) and SpA.^16,17^

Despite the progress in the treatment of axSpA, a significant proportion of patients do not respond adequately or are intolerant to TNFα and/or IL17 inhibitors.^18,19^ Therefore, drugs with alternative mechanisms of action are needed for the management of those patients. The lack of response to a single anti-cytokine therapy in SpA could be overcome by targeting a combination of cytokines. Given the paradigm of RA, targeting JAKs could be an effective approach for SpA, as well.^14^ Data from clinical studies reveal that JAK inhibition offers clinically significant improvements in skeletal and extra-skeletal SpA manifestations with an acceptable safety profile. In the following lines, clinical data on the use of JAK inhibitors in axSpA will be presented and the prospects created by their introduction in axSpA therapeutics will be discussed.

## EFFICACY OF JAK INHIBITORS IN AXSPA

### Ankylosing Spondylitis

#### Tofacitinib

**Figure 1.**
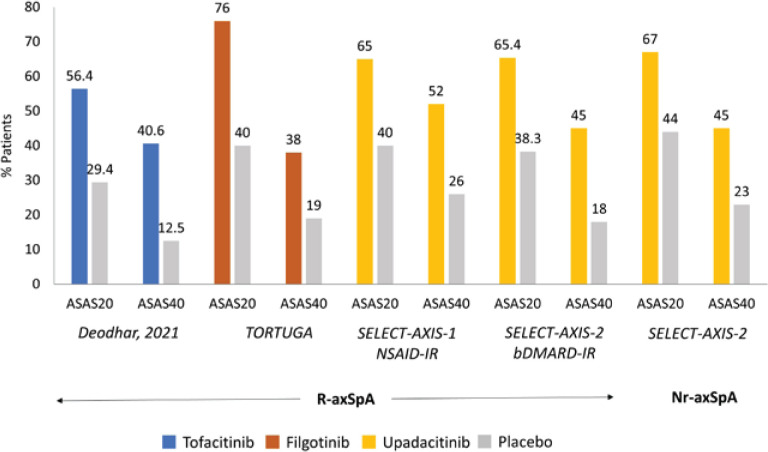
Efficacy of JAK inhibitors in latest clinical trials in axial spondyloarthritis: ASAS20 and ASAS40 responses are shown for filgotinib at 12 weeks, for upadacitinib at 14 weeks and for tofacitinib at 16 weeks in the respective trials. The data do not represent a head-to-head comparison, as they are taken from different studies. *ASAS: Assessment of SpondyloArthritis international Society; R-axSpA: radiographic axial spondyloarthritis; Nr-axSpA: non-radiographic axial spondyloarthritis

Tofacitinib is a non-selective inhibitor that inhibits JAK1, JAK3 and JAK2 in order of potency.^20^ Following a successful phase 2 study, a phase 3 clinical trial randomized patients with ankylosing spondylitis (AS) who were either naïve to bDMARDs (77%) or TNFα inhibitor inadequate responders (TNFi-IR, 22%) to tofacitinib 5 mg twice per day or placebo. At 16 weeks, tofacitinib-treated patients achieved Assessment of SpondyloArthritis Ιnternational Society 20% improvement (ASAS 20), the study primary endpoint, at a significantly higher proportion than those treated with placebo (56.4% *vs* 29.4%, p<0.0001) (**Figure 1**).^21^ Higher levels of clinical response, such as ASAS 40 and ASAS partial remission, were also achieved more frequently with tofacitinib and sustained in the long term during the study open label extension (**Table 1**). Regarding other efficacy endpoints, tofacitinib was associated with a rapid alleviation of pain and improvements across several patient-reported outcomes including fatigue, health-related quality of life and work productivity in both its phase 2 and 3 studies.^22,23^ Magnetic resonance imaging (MRI) data collected during the phase 2 study revealed that approximately one third of tofacitinib-treated patients achieved minimally important changes in the SPondyloArthritis Research Consortium of Canada (SPARCC) spinal and sacroiliac joint score.^24^ These data led to the Food and Drug Administration (FDA) approval of tofacitinib as a treatment for patients with active AS with previous failure or intolerance to at least one TNFα blocker^25^ and the recent European Medicines Agency (EMA) approval for the treatment of adult patients with active AS who have responded inadequately to conventional therapy.^26^

**Table 1.** ASAS responses with JAK inhibitors in Phase 3 clinical trials in axSpA.

**Disease**	**Study**	tsDMARD*	**Population studied**	**Primary endpoint**	ASAS 20 response to tsDMARD at week 12/14/16** (%)	ASAS 20 response to PBO at week 12/14/16** (%)	ASAS40 response to tsDMARD at week 12/14/16** (%)	ASAS40 response to PBO at week 12/14/16**(%)	**ASAS20 response to tsDMARD at OLE (%)**	**ASAS40 response to tsDMARD at OLE (%)**
AS	Deodhar A, 2021^21^	Tofacitinib	IR/intolerance to ≥2 NSAIDs or IR to ≤2 TNFi or prior bDMARD (TNFi or non-TNFi) use without IR (23%)	ASAS20 at week 16	56.4	29.4	40.6	12.5	at week 48^a^ 65.4	at week 48^a^ 50.4
	TORTUGA^27‡^	Filgotinib	IR/intolerance to ≥2 NSAIDs or previous use of ≤1 TNFi (9.5%)	Change of ASDAS from BL to week 12	76	40	38	19	n/a	n/a
	SELECT-AXIS 1^30†^	Upadacitinib	IR/intolerance to ≥2 NSAIDs/contraindication to NSAIDs	ASAS40 at week 14	65	40	52 (NRI) 54 (observed)	26	at week 64^31 b^ 79.6 (NRI) 93.7 (observed)	at week 64^31 b^ 72 (NRI) 84.8 (observed)
	SELECT-AXIS 2^34^	Upadacitinib	IR/intolerance to ≤2 bDMARDs (TNFi or non-TNFi)	ASAS40 at week 14	65.4	38.3	45	18	n/a	n/a
nrAxSpA	SELECT-AXIS 2^37^	Upadacitinib	IR/intolerance to ≥2 NSAIDs/contraindication to NSAIDs or IR/intolerance to 1 bDMARD (TNFi or IL17i) (33%)	ASAS40 at week 14	67	44	45	23	n/a	n/a

* In approved dosing regimen when applicable;

** based on the primary endpoint;

† phase 2/3 study;

‡ phase 2 study;

a continuous tofacitinib;

b continuous Upadacitinib; axSpA: axial spondyloarthritis; ASAS: Assessment of SpondyloArthritis international Society; ASAS20: ASAS ≥20% improvement; ASAS40: ASAS ≥40% improvement; ASDAS: Ankylosing Spondylitis Disease Activity Score; bDMARD: biologic disease-modifying antirheumatic drug; BL: baseline; IL17i: ΙL-17 inhibitor; IR: inadequate response; n/a: not available; NRI: non responder imputation; NSAIDs: non-steroidal anti-inflammatory drugs; OLE: open-label extension; PBO: placebo; TNFi: TNFα inhibitor; tsDMARD: targeted synthetic disease-modifying antirheumatic drug.

#### Filgotinib

A phase 2 study of the selective JAK1 inhibitor filgotinib in patients with active AS and inadequate response or intolerance to at least two NSAIDs or having previously received no more than one TNFα inhibitor (TORTUGA trial) was successful across a wide range of disease parameters (**Table 1**, **Figure 1**).^27^ Filgotinib at a daily dose of 200mg led to a significantly higher reduction from baseline in Ankylosing Spondylitis Disease Activity Score (ASDAS) at week 12, compared to placebo (−1.47 vs −0.57, p<0.0001). Significant improvements in disease activity with filgotinib were observed as early as 1 week. Bath Ankylosing Spondylitis Metrology Index (BASMI) also improved significantly with filgotinib compared to placebo by week 12 (−0.75 *vs* −0.39, p=0.0093). Regarding imaging, significant improvements in SPARCC MRI spine and sacroiliac joint scores at week 12 were seen in the filgotinib group compared with the placebo group (−5.7 *vs* 0.52, p=0.0066 and −3.52 *vs* 0.06, p=0.015, respectively). In a post-hoc analysis of TORTUGA, spine MRIs were assessed using the Canada–Denmark (CANDEN) MRI scoring system. Changes from baseline to week 12 in total spine score and subscores for inflammation, fat, erosion, and new bone formation at various anatomical locations were evaluated.^28^ Filgotinib significantly reduced spinal inflammation, particularly in the facet joints and posterolateral elements. Another post-hoc analysis of the trial revealed that SPARCC MRI sacroiliac joint erosion significantly decreased and backfill increased with filgotinib compared to placebo (p=0.02 and p=0.005, respectively) by week 12, suggesting that tissue repair starts early after treatment initiation.^29^

#### Upadacitinib

Upadacitinib, a selective JAK1 inhibitor, was investigated for the treatment of AS patients who had an inadequate response or intolerance to NSAIDs in the randomized, placebo-controlled phase 2/3 SELECT-AXIS 1 study.^30^ The study met its primary endpoint with 52% of patients in the 15mg once daily upadacitinib group achieving ASAS40 at week 14 compared to 26% in the placebo group (p=0.0003) (**Table 1**, **Figure 1**). Upadacitinib additionally led to a significantly greater change of the mean ASDAS compared to placebo (−1.45 vs −0.54 respectively at week 14, p<0.0001). Moreover, Bath Ankylosing Spondylitis Disease Activity Index 50 (BASDAI50) response rates were significantly higher for upadacitinib than placebo (45% vs 23% at week 14%, p=0.0016), as well as the Maastricht Ankylosing Spondylitis Enthesitis Score (MASES). Consistent significant improvements were also seen for mobility (BASMI), as well as AS quality of life score (ASQol) and AS Health Index. MRI assessment at week 14 also favoured upadacitinib over placebo with a treatment difference for SPARCC MRI spine score −6.71 [95% confidence interval (CI) −9.01 to −4.41, p<0.0001] and for SPARCC MRI sacroiliac joint score −3.91 (95% CI −5.05 to −2.77, p<0.0001). The one year open-label extension revealed sustained efficacy both for the patients initially randomized to upadacitinib and those who switched from placebo.^31^ Moreover, a post hoc analysis of SELECT-AXIS 1 showed a rapid (as early as week 2) and sustained -over 1 year- improvement in multiple pain-related outcomes including spine-specific ones, such as total and nocturnal back spinal.^32^ At 2 years, radiographic progression was minimal with 89.7% of patients showing an mSASSS increase <2 and 76.5% no mSASSS increase at all, respectively.^33^ SELECT-AXIS 2 (NCT04169373) was a clinical trial program which tested upadacitinib in two different axSpA populations: patients with active AS despite treatment with up to two bDMARDs (82% had been exposed to TNFi only) and patients with non-radiographic axSpA (nr-axSpA, see below). Concerning AS refractory to bDMARDs, the trial achieved its primary outcome, that is significantly more upadacitinib-treated patients attained ASAS40 at week 14 compared to patients receiving placebo (45% vs 18%, p<0.0001)(**Figure 1**).^34^ Similar to SELECT-AXIS 1, upadacitinib was significantly better than placebo in an array of outcomes, including ASDAS, total and nocturnal back pain, MASES, BASMI, ASQol, AS Health Index and SPARCC MRI spine and sacroiliac joint scores. Based on the above trial data upadacitinib has been approved by EMA for the treatment of active AS with inadequate response to conventional therapy^35^ and by FDA for patients with active AS and an insufficient response or intolerance to at least one TNFi.^36^

## NON-RADIOGRAPHIC AXSPA

As mentioned above, the SELECT-AXIS 2 protocol included the only study of JAKis in patients with nr-axSpA and objective evidence of inflammation as shown by increased serum C-reactive protein or a positive sacroiliac joint MRI. Included patients had failed NSAIDs or a bDMARD (33%). Significantly more nr-axSpA patients on upadacitinib 15mg daily achieved the trial primary endpoint, ASAS40 at week 14 compared to placebo (45% versus 23%, p<0.0001) (**Table 1**, **Figure 1**). Patients receiving upadacitinib showed significantly greater improvements in ASDAS, total and nocturnal back pain, MASES, ASQol, AS Health Index and SPARCC MRI spine and sacroiliac joint scores at 14 weeks of treatment.^37^ Based on these results, upadacitinib is the first JAKi approved for nr-axSpA by both EMA and FDA on the same conditions as in AS.^38^

## EFFICACY OF JAK INHIBITORS IN AXIAL PSORIATIC ARTHRITIS

Although it is still unclear whether AS and PsA with axial involvement are two distinct entities with overlapping features or parts of the same disease spectrum, in clinical practice they share several common therapeutic modalities.^39,40^ The trials of JAKis for PsA included patients with peripheral involvement.^41–46^ While both tofacitinib and upadacitinib are already approved for the treatment of PsA, the efficacy of JAKis in axial PsA largely remains unknown. It was recently reported that 23.1% and 27.5% of the patients with active PsA included in SELECT-PsA 1^44^ and SELECT-PsA 243 trials of upadacitinib in PsA had axial involvement, defined by both investigator assessment and patient reported outcome- (BASDAI ≥4 and BASDAI Question 2 ≥4 at baseline) based criteria.^47^ These patients demonstrated statistically greater clinical responses related to their axial involvement with upadacitinib 15 mg compared to placebo, and consistently numerically higher responses compared to adalimumab at week 24. A retrospective analysis of pooled data from clinical studies assessed the performance of JAK inhibitors in PsA in comparison with AS.^48^ Five publications in PsA were included (4 with tofacitinib and 1 with filgotinib). The tofacitinib studies showed that the drug significantly improved ASQol in PsA patients, although other AxSpA-specific measures were not available to analyse. Currently, there is a single ongoing phase 2 randomised double-blind, placebo-controlled study, PASTOR, which will assess the efficacy of tofacitinib in reducing MRI inflammation of the sacroiliac joints and the spine in patients with active axial PsA.^49^

## SAFETY

Since JAKis may be associated with effects in normal immune pathways beyond those targeted in disease, their safety is under continuous surveillance. The overall safety profile of JAKis in clinical trials has mainly been assessed in patients with RA and seems to be comparable to that of biologic cytokine inhibitors, except for an almost 2-fold higher risk for herpes zoster.^50,51^

The main safety issue of JAKis, however, concerns cardiovascular (CV) adverse events (AEs) and first emerged during the approval process of baricitinib for RA.^52^ As a result, the regulatory authorities requested additional studies enrolling RA patients with high CV risk, in order to compare the safety of tofacitinib (ORAL surveillance trial) and baricitinib (NCT03915964) with TNFα blockers. The publication of the results of ORAL surveillance showed that the combined dosages of tofacitinib (ie, 5mg twice daily and 10mg twice daily) failed to meet the study endpoint of non-inferiority regarding major adverse cardiovascular events (MACE) and cancers compared to TNFi.^53^ Subsequently, authorities in the USA and Europe noted this risk in tofacitinib’s Summary of Product Characteristics. Moreover, the FDA issued a black-box warning for increased risk for MACE, cancers and mortality, which was also extended to baricitinib and upadacitinib, and limited the prescription of all 3 drugs to patients having failed at least 1 TNFi. Recently, EMA’s safety committee also recommended that JAKis be prescribed to patients at increased risk for CV events, thromboembolic events or cancer (including but not limited to those at least 65 years old and smokers) only in the absence of suitable alternatives and that reduced doses be used in those patients.^54^ On the other hand, a post-hoc analysis of ORAL Surveillance comparing RA patients with and without a history of atherosclerotic CV disease, suggested that in the latter group the absolute excess risk might be quite low, despite the presence CV risk factors.^55^ Moreover, no statistically increased risk of CV events or malignancies with tofacitinib were identified in patients with RA treated in the real-world cohort of the more recent STAR-RA study.^56,57^

Concerns regarding gastrointestinal perforation in patients treated with JAKis have been expressed, as such a risk has been shown to be numerically, but not statistically, higher in patients treated with tofacitinib than those treated with bDMARDs.^58^ However, more data are needed. Laboratory abnormalities, such as anaemia, elevated transaminase and creatine phosphokinase (CPK) levels, alterations in the lipid profile, and infections, such as herpes zoster, are often seen in patients treated with JAKis, especially in those aged ≥65years and are mentioned in the EULAR points-to-consider for the use of JAKis.^59^

As axSpA has an earlier onset in life than RA, axSpA patients are usually younger suggesting a lower CV risk and fewer comorbidities. Moreover, the concomitant use of glucocorticoids and csDMARDs is less common in ax-SpA compared to RA. However, the CV risk is known to be increased in axSpA as well^60,61^ while AxSpA patients may still use NSAIDs, which raise the risk for MACE and gastrointestinal AEs. An outline of safety data from JAKi trials in AxSpA follows below.

### Tofacitinib

In the phase 2 trial of tofacitinib in AS, AEs with tofacitinib 5mg or 10mg twice daily were similar between the 2 dosing regimens, (53.8% and 51.9% versus 43.1% with placebo), with nasopharyngitis and upper respiratory tract infection being the most frequently reported.^62^ Two serious AEs (SAEs) led to drug discontinuation: peripheral swelling and one herpes zoster infection for 5mg twice daily and 10mg twice daily, respectively. Over the 16-week double blind period of the phase 3 trial of tofacitinib 5mg twice daily in AS, AEs were reported in 54.9% of patients receiving tofacitinib and 51.5% of patients on placebo.^21^ During 48 weeks of the study, no deaths, MACE, opportunistic infections or thromboembolic events were reported. Five cases (1.9% of tofacitinib-treated patients) of herpes zoster infections were reported.

### Filgotinib

In the TORTUGA trial, the filgotinib and placebo group had the same total AE and infection rates. Nasopharyngitis was the most common AE.^27^ One case of serious lower respiratory tract infection and another of deep vein thrombosis were reported in the filgotinib arm. No malignancies or deaths were reported through 12 weeks of the study.

### Upadacitinib

In SELECT AXIS 1 trial, 62% of upadacitinib- and 55% of placebo-treated patients had AEs with CPK elevation being the most common.^30^ Through week 14, the upadacitinib and placebo group had one serious AE each (not classified as of special interest). At one year, no serious infections, MACE, venous thromboembolic events or deaths were reported. There were 5 cases of herpes zoster corresponding to 2.1 events per 100 patient- years.^31^ A similar safety profile for upadacitinib was shown throughout the 14-week double blind period of the SELECT AXIS 2 studies, except for in the bDMARD-refractory group a higher incidence of serious infections (mostly COVID-19) was noted.^34,37^

Meta-analyses of safety data from phase 2 and 3 studies of JAKis in AS concluded that the incidence of AEs, serious AEs and treatment discontinuations did not differ between JAK inhibitors and placebo.^63,64^ Furthermore, no new safety signals, such as inflammatory bowel disease (IBD), new-onset uveitis or psoriasis, have been identified so far. Importantly, more safety data on the use of JAKis in patients with AxSpA are needed from long-term extensions of randomized trials and real-world observational studies to inform the safety profile of JAKis in patients with axSpA.

## DISCUSSION

Over the last decade, JAK inhibitors have emerged as a new class of drugs for the management of immune-mediated diseases and have received approval for a number of indications.^65^ The pathogenesis of axSpA unfolds through a complex network of inflammatory processes, some of which involve signalling through the JAK-STAT pathway, that makes axSpA a disease amenable to JAK inhibition.^66^ Given the previous experience with JAKis in RA, the need for new treatments for axSpA led to clinical trials of three compounds, tofacitinib, upadacitinib and filgotinib to assess their efficacy and safety in axSpA. The patient population most studied were those with radiographic axSpA who had failed NSAIDs, although most trials also included a small proportion of inadequate responders to bDMARDs. These trials yielded favourable results across several clinical domains of radiographic axSpA, including axial symptoms, enthesitis and a large set of patient-reported outcomes. Among them, the early and sustained pain relief stands out, as has been noted with JAKis in RA as well, and merits further investigation.^67^ The recent publication of the SELECT AXIS 2 trials of upadacitinib in patients refractory to bDMARDs and patients with nr-axSpA, completed the picture by demonstrating efficacy over the whole spectrum of axSpA.

Although JAK-STAT signalling may interfere with the IL-23/IL-17 axis and perhaps with other pathways governing bone turnover,^68^ the effects of JAKis on ax-SpA structural progression remains to be investigated. Although early MRI data support a positive role,^24,28–30^ the question remains as how clinically relevant these findings on MRI are, particularly regarding the long-term radiographic outcome.

The safety profile of JAKis in axSpA, as shaped by randomised clinical trials, appears acceptable. However, as JAKis are a relatively new drug class, long-term safety data still accumulate deriving mainly from the RA population. Although the ORAL Surveillance study in RA yielded a safety signal regarding malignancies and MACE,^53^ several meta-analyses of JAKi clinical trials did not reveal increased risks for thrombotic events compared to placebo,^50,69,70^ except for a baricitinib dose-dependent effect.^69^ Since axSpA patients are generally younger, with fewer comorbidities and less exposure to concomitant glucocorticoids and csDMARDs, they may be at less risk than their RA counterparts. However, awaiting more clinical trial and real-world data, a careful assessment of the cardiovascular risk profile of every axSpA patient should be made before a JAKi is prescribed. Accordingly, established cancer prevention strategies should be followed and a raised awareness for early signs of cancer should be demonstrated when dealing with patients on JAKi. A safety advantage of JAKi over biologics is the short half-life (usually spanning a few hours) which allows for the drug to be eliminated from the body within a few days in case of an adverse event, particularly infectious.^71^

No new safety signals were identified in the RCTs in AS, including cases of IBD, new onset uveitis or psoriasis. This is particularly relevant for patients with axSpA and co-existent IBD, since the treatment options for this patient group is limited to monoclonal anti-TNFα antibodies.^72^ Moreover, tofacitinib and upadacitinib are efficacious in ulcerative colitis and are already approved in this indication.^73,74^ Furthermore, upadacitinib and filgotinib have shown favorable results in Crohn’s disease in phase 2 studies,^75–77^ while the results of a phase 3 study of upadacitinib in Crohn’s disease are awaited. Therefore, tofacitinib and upadacitinib may represent alternative treatment options for patients with active axSpA despite anti-TNFα treatment and concomitant IBD. Apparently, such a decision should be discussed and agreed with a gastroenterologist, as well.

Choosing the best JAKi based merely on pharmacological terms is a challenging and likely misleading task. Indeed, predicting the efficacy of a JAK inhibitor in any disease is difficult considering the diversity of inflammatory and parenchymal cells which are involved in a vast array of pro-inflammatory and anti-inflammatory interactions, as well as differences among compounds regarding pharmacodynamics at the level of cytokine/receptor/cell, JAK selectivity and pharmacokinetics due to renal/hepatic factors, active metabolites etc.^3^ Therefore, pharmacologic properties should be assessed along with clinical efficacy, safety data and the patient profile, so as to choose the most suitable -if possible- JAKi. Regarding their position in the treatment of axSpA, clinical trial data support the use of tofacitinib and upadacitinib both after NSAID failure, as well as after inadequate response to bDMARDs. The 2019 ACR recommendations for the treatment of axSpA designated tofacitinib as an advanced-line therapy after TNFα and IL-17 inhibitors, with the reservation that the part regarding JAKis may change, since results from large clinical trials were still pending by then.^78^ In the updated ASAS-EULAR recommendations for the management of axSpA, TNFα, IL-17 and JAK inhibitors were equally included as a second-line treatment after NSAID failure, noting though that current practice are TNFα and IL-17 inhibitors.^79^ Finally, in the most recent GRAPPA recommendations JAKis may be considered for psoriatic axial disease as a second line after NSAIDs, along with TNFα and IL-17 inhibitors.^80^

In conclusion, JAK inhibitors have formally entered the therapeutics of axSpA. They are effective over a broad spectrum of disease manifestations, both skeletal and extra-skeletal. They have a fast onset and sustained action, including pain relief, they are easily taken via the oral route and have a safety profile compatible with what has already been observed in RA. Therefore, they represent a useful treatment option for axSpA patients, while, conversely, their clinical application will likely help further elucidate the pathophysiology of the disease. More research is needed to identify the best patient candidates for JAKi treatment and to establish the long-term safety and efficacy profile, including the effects in structural progression.
